# Diagnostic Value of the Combined Measurement of Serum Hcy, Serum Cys C, and Urinary Microalbumin in Type 2 Diabetes Mellitus with Early Complicating Diabetic Nephropathy

**DOI:** 10.1155/2013/407452

**Published:** 2013-09-18

**Authors:** Tengkai Wang, Qian Wang, Zhimei Wang, Zuomin Xiao, Lunqin Liu

**Affiliations:** ^1^Department of Clinical Medicine, Taishan Medical College, Tai'an, China; ^2^Department of Clinical Medicine, Shandong University School of Medicine, Ji'nan, China; ^3^Department of Laboratory Medicine, Shandong University Qilu Hospital, Ji'nan 250012, China; ^4^Department of Laboratory Medicine, The Third People's Hospital, Ji'nan, China; ^5^Department of Laboratory Medicine, Ji'nan Iron and Steel Group Corporation General Hospital, Ji'nan, China

## Abstract

Diabetic nephropathy (DN) is a major cause of end-stage kidney disease, and therefore early diagnosis and intervention may help reverse renal damage. One hundred and sixty-eight patients with T2DM and 56 healthy volunteers (control group) were enrolled at Shandong University Qilu Hospital between April 2010 and October 2012. All subjects underwent blood sampling for sera homocysteine (Hcy) and cystatin C (Cys C) assays and a urine microalbumin test. The patients were divided into three groups according to the urine microalbumin excretion rate (UMAER): the simple DM group (SDM group, *n* = 51), the early-stage DN group (EDN group, *n* = 60), and the clinical DN and renal failure group (CDN group, *n* = 57). Correlation analysis was performed to examine the association between sera Hcy and Cys C levels with UMAER. Our findings showed that sera Hcy level, Cys C level, and UMAER increased significantly in the SDM group (*P* < 0.05, *P* < 0.01), the EDN group (*P* < 0.01), and the CDN group (*P* < 0.01) as compared with the control group. These three biochemical markers also increased significantly with DN progression (*P* < 0.01). Correlation analysis showed that sera Hcy and Cys C levels were positively correlated with UMAER (*r* = 0.702, *P* < 0.01; *r* = 0.873, *P* < 0.01). In conclusion, our results showed that sera Hcy and Cys C levels increased consistently with the development and progression of DN as indicated by UMAER. Sera Hcy and Cys C are sensitive biomarkers for the detection of early-stage DN and monitoring its progression.

## 1. Introduction

Diabetic nephropathy (DN) is a major microvascular complication of diabetes mellitus. Renal damage may underlie early-stage DN, which, in most cases, is asymptomatic. Proteinuria is present in patients with advanced stage DN. Early detection and intervention in diabetic patients with DN will possibly reverse, or even eliminate, underlying renal damage. Therefore, the early diagnosis and treatment of DN are crucial [1, 2]. The urine microalbumin excretion rate (UMAER) is a traditional standard measure for evaluating glomerular filtration and is helpful for detecting early-stage DN and monitoring its progression. However, a combination of biomarkers is needed to improve the prediction of DN in clinical practice. 

Recent studies have suggested that DN is a chronic inflammatory disorder mediated by a series of cytokines, including interleukin-6, interleukin-8, and tumor necrosis factor-*α* as well as homocysteine (Hcy) and cystatin C (Cys C). Homocysteinemia is an independent risk factor for cardiovascular diseases [3] and has been reported in patients with chronic kidney diseases [4]. Hcy is thought to be associated with microvascular complications in diabetic patients [5–8]. As a member of the cysteine protease inhibitor superfamily involved in the metabolism of Hcy, Cys C is an ideal endogenous marker for estimating glomerular filtration and identifying early-stage glomerular damage. The recent study suggested that Cys C can also be used as predictor of heat failure [9–11]. The mAlb in urine had been known as an indicator for evaluating glomerular filtration and used for the early diagnosis and stage of DN.

The primary objective of this study was to investigate whether sera Hcy and Cys C levels are associated with UMAER in patients with DN. The usefulness of sera Hcy and Cys C assays combined with UMAER for detecting early-stage renal damage in diabetic patients was also determined. 

## 2. Materials and Methods

### 2.1. Patient Recruitment

The study protocol was approved by the Institutional Review Board at Shandong University Qilu Hospital. One hundred and sixty-eight patients, who were diagnosed with T2DM in accordance with the World Health Organization Diagnostic Criteria for Diabetes Mellitus [12], were enrolled between April 2010 and October 2012. The staging of DN was determined as previously reported [13]: stage I-II (simple DM (SDM)) with a UMAER <30 mg/24 h was observed in 51 patients (24 males and 27 females), aged 48.2 ± 12.6 (range, 39–72); stage III (EDN) with a UMAER of 30–300 mg/24 h was observed in 60 patients (28 males and 32 females), aged 51.3 ± 13.4 (43–75); and stage IV-V (CDN) with a UMAER >300 mg/24 h was observed in 57 patients (27 males and 30 females), aged 54.0 ± 14.1 (46–76). Fifty-six healthy volunteers (26 males and 30 females) without a known history of DM, aged 49.2 ± 13.7 (40–74), were simultaneously recruited as controls. All groups were comparable in terms of age and sex. Concomitant cardiovascular, hepatic, or other medical conditions were excluded in all subjects. None of the subjects had received folic acid, vitamin B, anticoagulants, or antiplatelet agents within 2 months prior to enrollment. All patients gave written informed consent prior to participation in this study.

### 2.2. Urine and Blood Sampling

All subjects were instructed to collect 24 h urine samples (from 07.00 am on the day) in a clean plastic ware containing 10 mL xylene. A well-mixed urine sample (5 mL) was used for the UMAER test. Fasting venous blood (3 mL) was sampled and centrifuged at 3,000 rpm for 10 min, and the sera were stored at −80°C for Hcy and Cys C assays.

### 2.3. Clinical Biochemistry Assays

Urinary microalbumin was measured using an automatic immunoturbidimetric assay (Siemens Healthcare Diagnostics Products GmbH, Marburg, Germany) with a BN ProSpec automatic protein analyzer (Dade Behring Marburg GmbH, Marburg, Germany). An enzyme-linked immunosorbent assay kit (Baoman Biotechnology Co., Ltd., Shanghai, China) was used to measure urinary microalbumin at a concentration below 10 mg/L. Serum Hcy was measured using an electrochemiluminescence immunoassay (Hitachi High-Technologies Corporation, Tokyo, Japan) with a Roche Cobas E601 automatic electrochemiluminescence analyzer (Roche Diagnostics Co., Ltd., Mannheim, Germany). Serum Cys C was measured using a latex-particle-enhanced immunoturbidimetric assay (Ailex Scientific, Co., Ltd., Shanghai, China) with an Olympus 2700 automatic biochemistry analyzer (Olympus Corporation, Tokyo, Japan). 

### 2.4. Statistical Analysis

SPSS 13.0 statistical software (SPSS Inc., Chicago, IL, USA) was used for statistical analysis. Continuous data were expressed as mean ± SD, and the means between two groups were compared using Student's independent two-sample *t*-test. Correlation analysis of sera Hcy and Cys C levels with UMAER was performed using a linear correlation model. 

## 3. Results

### 3.1. Sera Hcy and Cys C Levels Increase with DN Progression in Diabetic Patients

The results of sera Hcy and Cys C assays and the UMAER test in diabetic patients and control subjects are shown in Table 1. Sera Hcy and Cys C levels and UMAER were significantly increased in the SDM group (*P* < 0.05, *P* < 0.05 and *P* < 0.01, resp.), the EDN group (*P* < 0.01, *P* < 0.01 and *P* < 0.01, resp.), and the CDN group (*P* < 0.01, *P* < 0.01 and *P* < 0.01, resp.) as compared to the control group. In diabetic patients, all three markers were elevated more in the EDN group than in the SDM group (all *P* < 0.01). These significant increases were also seen in the CDN group as compared to the EDN group (all *P* < 0.01).

### 3.2. Correlation Analysis

Correlation analysis of sera Hcy and Cys C (dependent variables) against UMAER (independent variable) showed that both Hcy and Cys C levels were positively correlated with UMAER (*r* = 0.702, *P* < 0.01; *r* = 0.873, *P* < 0.01, resp.). 

## 4. Discussion

Glomerular filtration rate (GFR) is believed to be the best indicator of renal function. The clearance rate of exogenous substances, such as inulin and radioisotopes, is considered to be the standard estimation of glomerular filtration. However, this method is frequently limited by access to equipment and an eligible population, and the administration of exogenous substances also poses some safety risks to humans. It is likely that the clearance rate of endogenous substances, such as blood urea nitrogen and creatinine, would not reveal early-stage glomerular damage as the kidneys have a strong compensation function. Endogenous substance clearance may also be affected by protein intake, metabolic activity, and some drugs [14]. These routine tests are not effective in detecting early-stage renal damage in diabetic patients as DN normally has a latent onset. Therefore, laboratory diagnostic markers are desired for the prediction and early diagnosis of DN in clinical practice.

Microalbumin, a large molecular-weight protein, leaks through the glomerular membrane into urine only when glomerular filtration is disrupted. Urinary microalbumin is a major marker of damage to the charge selectivity and barrier integrity of the glomerular membrane. Therefore, the UMAER is considered the standard measure of glomerular damage. Normally, urine contains a very low concentration of albumin. However, urinary albumin concentration increases if the leakage of albumin through the glomerular membrane is beyond reabsorption by the renal tubule, indicating serious glomerular damage. Chronic hyperglycemia and poor blood glucose control will thicken the glomerular basal membrane, resulting in glomerular hyperinfiltration, and increase microalbumin leakage into the urine. Our results showed that even asymptomatic SDM patients had a significantly increased UMAER. This measure can be used to determine the severity and staging of DN in diabetic patients. The UMAER test is a relatively simple procedure, but it requires the collection of 24 h urine samples. Microalbumin excretion may also be affected by position, exercise, urinary tract infection, stress response, and protein intake.

Hcy is a major metabolite of methionine and cysteine, and this metabolite is derived mainly from remethylation of methionine. Hyperhomocysteinemia has been shown to be associated with a series of chronic, noninfective diseases, such as coronary heart disease, diabetes mellitus, and stroke, and has been increasingly reported as an independent risk factor of DN [15–17]. Serum Hcy level is also an independent factor affecting UMAER [18]. Hyperhomocysteinemia exerts a mutual effect on renal damage occurring in DN patients, as Hcy is primarily synthesized and metabolized in the kidneys [19]. Both a reduction in GFR and early-stage tubular epithelial disorder can increase serum Hcy level. Our results showed that Hcy increased in SDM patients as compared to control subjects and continued to increase with DN progression. This may have been due to Hcy metabolism abnormalities caused by insulin resistance or insufficiency. Fonseca et al. [20] reported that the insulin sensitivity index was negatively correlated with plasma Hcy level. This finding suggests that Hcy may be a predictive factor for DN, and regular monitoring of serum Hcy level in diabetic patients may help detect early-stage DN and prevent the occurrence and progression of renal damage. The mechanism underlying hyperhomocysteinemia-associated DN is unknown. It is possible that Hcy is easily oxidized by the blood into toxic superoxide anions and hydroxyl radicals [21, 22]. Excess generation of these oxidants will compromise the elimination of free radicals by microvascular endothelial cells and initiate cell membrane lipid peroxidation. Membrane integrity damage will consequently result in microvascular endothelial impairment and degeneration. Hyperhomocysteinemia may lead to microvascular thrombosis by activating oxidative stress, direct cytotoxicity, the nitric oxide pathway, and coagulation factors [22–24]. The formation of microvascular thrombi will damage endothelial cells and impair glomerular filtration.

Cys C, also called *γ*-microprotein, is a homocysteine protease inhibitor synthesized by various nucleated cells. This microprotein is a low-molecular weight, basic, and nonglycosylated protein widely present in nucleated cells and humoral fluids throughout the body. The synthesis of Cys C is normally constant and less likely to be affected by age, sex, nutritional status, inflammatory response, or diet. Circulating Cys C can only be filtered by the glomerular membrane and is almost completely reabsorbed and metabolized by the proximal convoluted tubule without returning to the circulation. Tubular epithelial cells do not secrete Cys C. Even minimal glomerular damage will result in a significant increase in serum Cys C level, causing disease progression. Therefore, Cys C is thought to be a more sensitive and specific endogenous substance for estimating GFR [25, 26]. As a specific homocysteine protease inhibitor, Cys C can inhibit Hcy degradation and increase serum Hcy level. Cys C will synergize with Hcy and cathepsin and impact adversely on microvascular endothelia [18]. Our results showed that the great majority of diabetic patients had a significant increase in serum Cys C. The serum levels of both Hcy and Cys C increased more significantly in CDN patients as compared to EDN and SDM patients and showed a positive correlation with UMAER. This suggests that Hcy and Cys C may contribute synergistically to the occurrence and progression of DN in diabetic patients. 

Sera Hcy and Cys C assays can be carried out using multiple automatic analysis systems, such as particle-enhanced luminescence or scattering immunoturbidimetry and fluorescence immunoassay. These analyses are easy to perform, less likely to be affected by confounding factors, and more reproducible as compared to the UMAER test. Sera Hcy and Cys C assays in combination with the UMAER test are expected to be more effective in detecting early-stage DN [27, 28].

In conclusion, sera Hcy and Cys C levels increased significantly with UMAER in diabetic patients as compared to control subjects. These levels were elevated more in DN patients compared to SDM patients. A positive correlation between sera Hcy/Cys C level and UMAER was observed. This suggests that the increases in sera Hcy/Cys C and urinary microalbumin are closely associated with the occurrence and progression of DN in diabetic patients. The levels of these three markers increased preceding the occurrence of proteinuria and even in the presence of minimal glomerular damage. Therefore, sera Hcy and Cys C assays in combination with the UMAER test are clinically useful for detecting early-stage DN and monitoring disease progression. This combined assay method will allow a more sensitive and accurate evaluation of renal damage in early-stage DN. Timely intervention for hyperhomocysteinemia and cystatinemia will be helpful in preventing and delaying the development and progression of DN.

## Figures and Tables

**Table 1:** Sera Hcy and Cys C levels and UMAER (mean ± SD) in diabetic patients and control subjects.

Group	*n *	Hcy (*μ*mol/L)	Cys C (mg/L)	UAER (mg/24 h urine)
Control	56	7.3 ± 4.0	0.9 ± 0.4	12.0 ± 6.4
SDM	51	9.4 ± 5.1*	1.1 ± 0.6*	19.4 ± 7.7**
EDN	60	15.5 ± 7.7^∗∗△^	2.0 ± 1.2^∗∗△^	141.9 ± 83.9^∗∗△^
CDN	57	28.7 ± 15.6^∗∗▲^	3.5 ± 1.7^∗∗▲^	584.7 ± 334.7^∗∗▲^

Hcy: homocysteine; Cys C: cystatin C; UMAER: urine microalbumin excretion rate; SDM: simple diabetes mellitus; EDN: early diabetic nephropathy; CDN: clinical diabetic nephropathy. *P < 0.05 and **P < 0.01 for diabetic patients versus control subjects; ^△^P < 0.01 for EDN versus SDM; ^▲^P < 0.01 for CDN versus EDN.
